# Combined Endoscopic Transorbital and Endonasal Repair of High Flow Orbital Apex/Middle Fossa Cerebrospinal Fluid Leak with a Nasoseptal Flap

**Published:** 2018-03-30

**Authors:** Brandon Lucke-Wold, Gustavo Mendez, David Cua, Paul Akins, Haley Gillham, Jeremy Ciporen

**Affiliations:** 1 Department of Neurosurgery, West Virginia University School of Medicine, USA; 2 Department of Radiology, Oregon Health and Science University, USA; 3 Department of Neurosurgery, The Permanente Medical Group, USA; 4 Department of Neurosurgery, Oregon Health and Science University, USA

**Keywords:** orbital CSF leak, Combined transorbital/endonasal repair, orbital exenteration, nasoseptal flap

## Abstract

**Background and importance:**

High flow orbital apex or middle fossa cerebrospinal fluid (CSF) leaks can be life threatening and complex to repair. These leaks associated with large dural defects are most commonly repaired with an open temporalis muscle patch or free flaps, but these flaps do not always stop the leak.

**Clinical Presentation:**

A 65-year-old patient presented two years after orbital exenteration and radiation for squamous cell carcinoma. He developed multi-organism meningitis and pneumocephalus secondary to a large high-flow orbital apex/middle fossa CSF leak. To repair the leak, a combined endoscopic transorbital/endonasal approach with pedicled nasospetal flap and dermis fat graft was used. We describe the unique endoscopic technique that was used to treat the life threatening high flow orbital apex/middle fossa CSF leak. The technique allowed the use of the transposed pedicled flap, which is an alternative to the free flap in controlling CSF leak. Cisternogram post-operatively and clinical exam confirmed resolution of CSF leak. Although a critically ill patient at admission with a modified Rankin scale (MRS) of 5, he was discharged home on continued IV antibiotic therapy with a MRS of 3. Endoscopic evaluation at three months after treatment showed the effectiveness of the flap and he continued to improve clinically.

**Conclusion:**

This is the first case to describe a combined endoscopic transorbital and endonasal repair of high flow orbital apex/middle fossa CSF leak with a pedicled nasoseptal flap. These techniques can be utilized during initial reconstruction after orbital exenteration or as a salvage flap.

## Introduction

### Background and importance

Cerebrospinal fluid (CSF) leak following oculoplastic surgery is rare but has potentially serious complications [1]. Orbital exenteration can expose a significant portion of the skull base and depending on the underlying surgical pathology, the dura could be compromised during surgical resection leading to CSF leak [2]. Once a leak does occur, 31% of patients have major complications [3]. The most serious of these is meningitis, which can be a life-threatening emergency. Due to the location and pre-existing pathology, non-traditional organisms such as pasteurella multocida can be the culprit for meningitis [4]. Patients often present with headache and visual changes but can quickly deteriorate clinically [5]. Another potential life-threatening complication is tension pneumocephalus [6]. It is therefore imperative that skull base reconstruction be performed, and aggressive management of the CSF leak be obtained [7]. Using traditional open techniques, a temporalis muscle patch or free flap is often needed. These flaps are limited in that the vascular supply is cut off and CSF leak commonly recurs [8]. Endoscopic techniques provide the ability to use vascularized flaps that are more reliable and durable for repairing the skull base defects and preventing recurrent CSF leak [9]. In this case report, we demonstrate the utility of using a combined endoscopic transorbital and endoscopic endonasal approach to repair a high flow orbital apex/middle fossa CSF leak.

## Clinical Presentation

**Figure 1:**
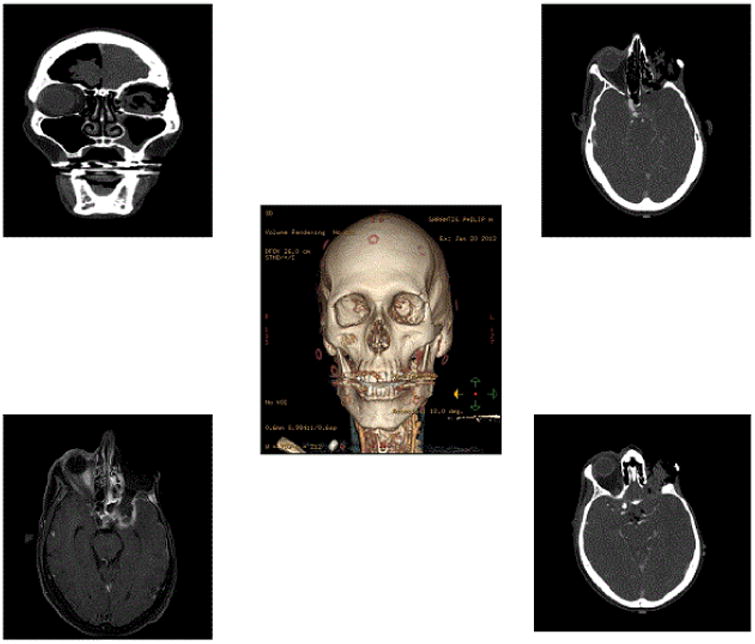
Pre-operative CT imaging showing pneumocephalus and signs of meningitis

Our patient is a 65 year-old-male who had a history of squamous cell carcinoma that was invading through the orbit. Two years prior to presentation he had extensive orbital exenteration and resection of the tumor. This was followed by radiation therapy. Unfortunately, he developed a high-flow orbital apex/middle fossa CSF leak (Figure 1).

**Figure 2:**
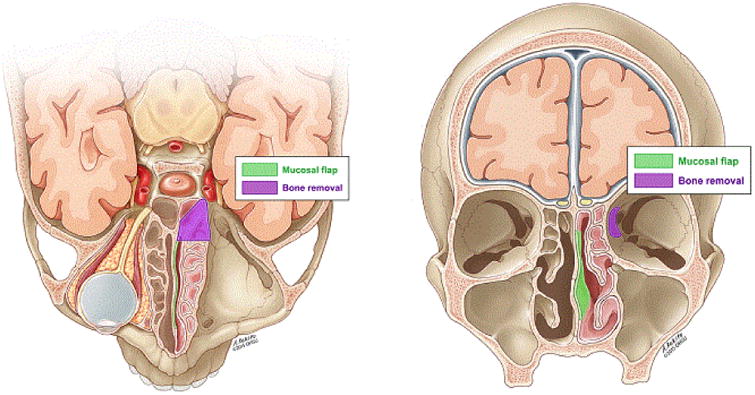
Illustrations showing visualization of flap that can be obtained with combined approach

This was complicated by multi-organism meningitis and pneumocephalus. At presentation to the hospital his modified Rankin scale (MRS) score was 5. It was decided to perform emergent surgery with a combined endoscopic transorbital and endoscopic endonasal approach. Otolaryngology and oculoplastic reconstructive surgeons were consulted. Given the temporalis atrophy, extent of radiation changes to the patient's head and neck, and prior surgeries a free flap, temporalis, or temporoparietal fascial flap (TPFF) were not viable options. The endoscopic approach was chosen to enhance the feasibility of using a pedicled vascular flap instead of a free flap. A transorbital port was made through the ethmoid bone and combined with a trans-nasal placed endoscope. This combination allowed ample visualization for transposing the nasoseptal flap to cover the anatomical defect (Figure 2). An abdominal dermis fat graft was placed over the temporal lobe/orbital defect. This provided volume and a template for the pedicled nasoseptal flap to heal over.

**Figure 3:**
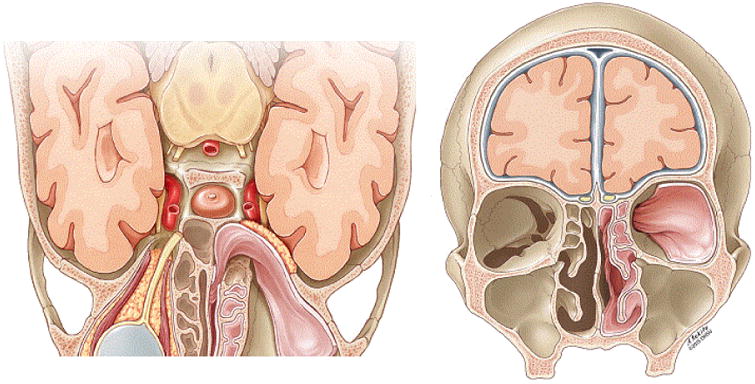
Illustration showing how pedicled nasoseptal flap is applied to orbital wall

**Figure 4:**
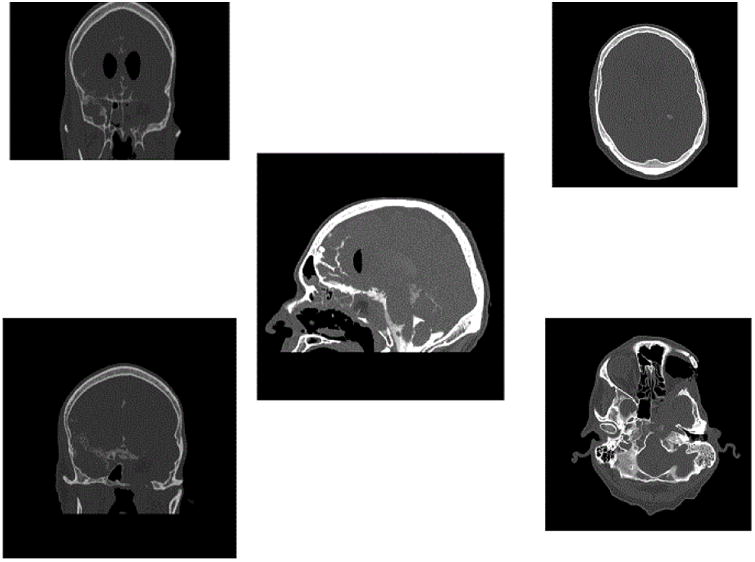
Post-operative CT cisternogram showing no CSF leak

**Figure 5:**
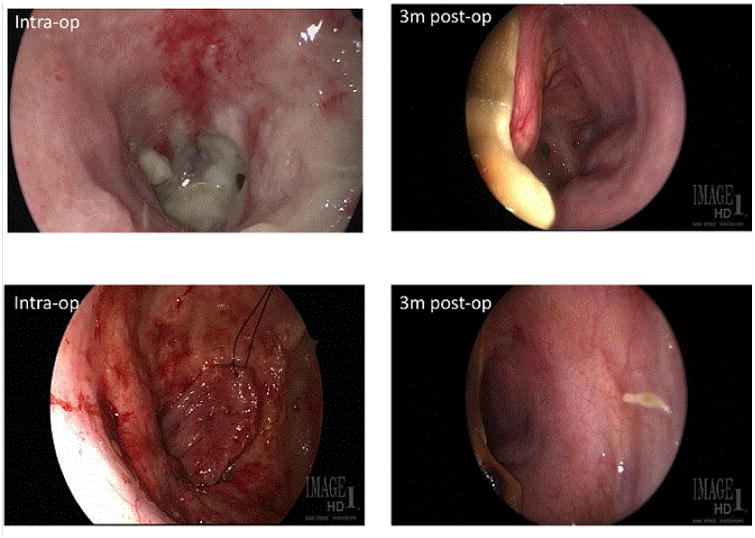
3-month endoscopic imaging compared to intraoperative imaging showing healing and continued vascularization of the flap

The nasoseptal flap was designed to be as long as it was wide. The inferior cut was along the nasal floor and the superior cut along the plane of the superior ostia of the left sphenoid ostia-approximately 1 cm inferior to the skull base. The vertical limb anteriorly was taken just shy of the columella. The left sphenoid osteotomy was performed and removal of the left side of the face of the sphenoid. The sphenoid sinus could be visualized from the transorbital approach. The flap was transposed anteriorly over the left carotid artery. It was ensured that the flap had some redundancy anticipating flap retraction. It has been proposed that the nasoseptal flap can contract approximately 30% and may lead to CSF leak if it pulls away from the site of repair [10]. The flap was placed over the orbital defect and the dermal fat graft. The patient had exposed lateral orbital bone. Tack up C1 bit was used to make wire pass holes. Through the wire pass holes in the lateral orbital wall, 4-neurolon sutures were used to maintain the position of the graft and minimize the risk of flap retraction and recurrent CSF leak. Abdominal fat was also used on the anterior aspect of the nasoseptal flap from the orbital approach to maintain the flaps contact with the dermal fat graft within the orbital defect, to promote healing, and minimize dead space. The abdominal fat graft was dressed with xeroform dressing. The abdominal fat graft reabsorbed over time and the nasoseptal flap healed very well. The flap transposition can be seen in (Supplementary file), and how it was secured down atop the orbital wall seen in (Figure 3). A lumbar drain was used for 6 days and clamped. A post-op cisternogram was done on postoperative day 7 and showed no CSF leak (Figure 4). The lumbar drain was removed after the cisternogram confirmed that there was no recurrent CSF leak. The patient was discharged on antibiotic treatment with an improved MRS of 3. Endoscopic evaluation at 3 months post-op showed excellent tissue healing and continued vascular supply to the transposed flap (Figure 5). The patient recovered well with resolution of the meningitis and pneumocephalus and no recurrence of CSF leak.

## Discussion

Although CSF leaks are relatively rare, tumors or foreign bodies that invade into the orbit are associated with higher occurrence of these leaks [11]. Once a leak does occur, the need for definitive repair is critical. Ramakrishna and colleagues showed the efficacy of using a transorbital endoscopic approach to repair these CSF leaks [12]. The limitation of the transorbital approach alone is that it is not sufficient for high flow CSF leaks with significant dura disruption. We present here the first case using a combined transorbital and endonasal approach. The combined approach increases visualization to allow manipulation of surgical instruments. By having the increased working space, a pedicled nasoseptal flap can be readily prepared and transposed. The pedicled nasoseptal flap is the workhorse flap for repairs and is especially suitable for high flow leaks [13]. The approach can be used at time of initial tumor resection or as a salvage repair. Palejwala and colleagues suggest that for large tumor resections the flap should be considered early [14].

In our patient, the CSF leak was associated with multi-organism meningitis and pneumocephalus. Meningitis can be life threatening in these patients and lead to rapid clinical decline [15]. Our patient already had a MRS of 5 at admission. Once meningitis is present, surgical intervention is necessary because conservative management has failed [16]. Pneumocephalus is often present in these cases and will require emergent decompression if signs of tension pneumocephalus are present and apparent on imaging [17]. To differentiate between tears and CSF, the fluid should be collected and tested for beta-2 transferrin [18]. If beta-2 transferrin is present, a spinal tap should be performed urgently, especially if the suspicion for meningitis is high. This will help guide the antibiotic regimen to be given perioperatively and post-operatively. The patient will need to be managed in the intensive care unit after surgery and carefully monitored for CSF leak recurrence. Fortunately, using the combined endoscopic technique the CSF leak was successfully managed, which allowed the meningitis to be treated with antibiotics. At three months after treatment, the flap had maintained its vascularity and the nasal passage was well healed.

## Conclusion

In conclusion, for high flow orbital apex or middle fossa CSF leaks the combined transorbital and endonasal endoscopic approach is ideal. The combined transorbital and endonasal endoscopic approach should be considered for all high flow orbital apex or middle fossa CSF leaks.

## Supplementary Material

Video 1

## Highlights

- Pedicled nasoseptal flap should be considered in refractory high flow orbital apex CSF leaks.
- Dermal fat graft should be considered to serve as a template upon which the vascularized flap to heal. This graft serves to lessen dead space especially for larger skull base defects. It would have been unlikely for a vascularized flap to heal against the exposed temporal lobe tissue itself.
- Nasospetal flap design for redundancy. Flaps on tension do not heal well. Therefore, redundancy is critical.
- Ensure the vascularized flap is not placed over mucosa for the flap will not heal effectively and may lead to recurrent CSF leak.
- Tacking sutures either to bone or soft tissue to maintain the flap position during healing and retraction period to minimize the risk of recurrent CSF leak due to flap retraction.
- Consider a pressure graft either autograft or allograft to maintain anterior pressure upon the vascularized flap to maintain contact with the bone edges and tissue template to minimize dead space and promote healing to the defect.
- Consider multi-portal and endoscopic approaches to the skull base for visualization and improved access to challenging to reach regions of the skull base via a single approach.
- Consider temporary CSF diversion external ventricular drain or lumbar drain to allow for wound healing.
- In cases in which pneumocephalus is present, the priority should be to seal the skull base defect. Use of an EVD or LD in the setting of a skull base defect leading to pneumocephalus may lead to tension pneumocephalus if the skull base defect is not sealed.
